# Case Report: Anti-NF186+ CIDP After Receiving the Inactivated Vaccine for Coronavirus Disease (COVID-19)

**DOI:** 10.3389/fneur.2022.838222

**Published:** 2022-03-14

**Authors:** Shirui Wen, Kailing Huang, Haoyue Zhu, Peihong Li, Luo Zhou, Li Feng

**Affiliations:** Department of Neurology, Xiangya Hospital, Central South University, Changsha, China

**Keywords:** inactivated COVID-19 vaccine, NF186, chronic inflammatory demyelinating polyneuropathy (CIDP), autoimmune disease, COVID-19

## Abstract

Corona Virus Disease 2019 (COVID-19), the novel coronavirus disease, is now a global pandemic. Vaccination can significantly reduce the mortality rate caused by the severe acute respiratory syndrome of coronavirus 2 (SARS-CoV-2). There are currently several effective vaccines that have been introduced. Inactivated COVID-19 vaccine is one of these options and is generally considered safe. Neurofascin (NF) plays an important role in keeping the functionality of the node of Ranvier. We report here a rare case of anti-NF186+ chronic inflammatory demyelinating polyneuropathy (CIDP) in a 23-year-old male patient who was vaccinated with inactivated COVID-19 vaccine prior to the onset. This report adds a new possible rare side effect of a COVID-19 vaccine and provides a case for the clinical effectiveness of rituximab (RTX) in patients with anti-NF186+ CIDP.

## Introduction

Since the end of 2019, the Corona Virus Disease 2019 (COVID-19) caused by the severe acute respiratory syndrome of coronavirus 2 (SARS-CoV-2) has posed a significant threat to the world. The disease is an acute severe respiratory syndrome with florid pulmonary manifestations and multi-organ involvement including cardiovascular, musculoskeletal, gastrointestinal, and neurological complications. Chronic inflammatory demyelinating polyneuropathy (CIDP) is one of the autoimmune disorders of the peripheral nervous system. Vaccination is the most crucial way to contain the COVID-19 pandemic. Inactivated or live-attenuated viruses, as well as recombinant proteins and vectors technologies, have been employed to develop the COVID-19 vaccine. So far, there is no case report of anti-NF186+ CIDP after exposure to the COVID-19 inactivated vaccine. This may be the first case of anti-NF186+ CIDP after receiving vaccination based on our knowledge.

### Case Reports

A 23-year-old male was admitted to the Department of Neurology, Xiangya Hospital, Central South University on June 22, 2021, with acute limb weakness and numbness for 28 days. He had received his second dose of inactivated coronavirus vaccine the day before these symptoms appeared. On May 26, 2021, the patient developed weakness of the left upper limb, and subsequently, the symptoms progressed to numbness and weakness of the extremities. On June 12, he went to the local hospital for the examination of cerebrospinal fluid (CSF), which showed increased total protein (0.99 g/L) and normal cell count (4 × 10^6^/L). He was initially diagnosed with Guillain-Barre syndrome (GBS). After the intravenous injection of a human immunoglobulin [0.4g/(kg d)] from June 16 to 20, there was no significant improvement in the symptoms but gradual aggravation occurred. Therefore, he came to our hospital for treatment. There was no history of any viral or respiratory illness before the symptoms appeared. His past medical history was received wherein it is stated that his first dose of inactivated coronavirus vaccine was on March 28, 2020, and the second dose was on May 25, 2021. There was nothing special about personal and family history.

Physical examination revealed symmetrical upper limb weakness [Medical Research Council (MRC) grade 3/5 distally and 2/5 proximally] and lower limb weakness (MRC grade 2/5 distally and 1/5 proximally) and areflexia. The long glove-like pain sensation below the elbow joint of both upper limbs and the knee joint of both lower limbs decreased. Bilateral pathological signs were not elicited.

### Auxiliary Examination

The patient's comprehensive examinations showed that the platelets were low, urine protein was positive, hematuria, complement C3, and C4 were significantly reduced, and many kinds of autoantibodies were positive, such as thyroid antibodies, rheumatic immune antibodies (see Supplementary Table 1). Sensory nerve conduction studies (NCS): upper limbs showed reduced sensory nerve action potential amplitudes; the lower limbs were normal. Motor NCS: upper limbs showed severely reduced compound muscle action potential amplitude responses and conduction velocities; the lower limbs showed reduced compound muscle action potential amplitude responses and normal conduction velocities. The tibial F wave and H reflex latencies were prolonged. Needle electromyogram (EMG) displayed neurogenic damage. The MRI of the brain and spine was normal and there was no evidence of central destination on T2 and fluid attenuated inversion recovery (FLAIR) images. Re-examination of cerebrospinal fluid analysis in our hospital showed elevated protein (0.86 g/L) and the normal level of leucocytes (3 × 10^6^ L). The patient was diagnosed with immune-associated peripheral neuropathy on admission, and GBS was most likely to be considered. The patient began to receive methylprednisolone pulse therapy.

In the course of treatment, the patient's limb weakness gradually aggravated, manifested as symmetrical limb weakness (MRC grade 0/5 distally and 0/5 proximally), and slowly involving the cranial nerves, like inadequate bilateral eyeball abduction, diplopia. On July 2, the patient's condition further deteriorated. He developed dyspnea with type II respiratory failure and was transferred to the intensive care unit. During the treatment, the tracheotomy ventilator was given to assist breathing, and the high dose of intravenous injection of glucocorticoid was gradually reduced to an oral low dose for maintenance. The patient received plasma exchange on July 9 and 14, respectively. However, his condition did not improve significantly.

**Figure 1:**
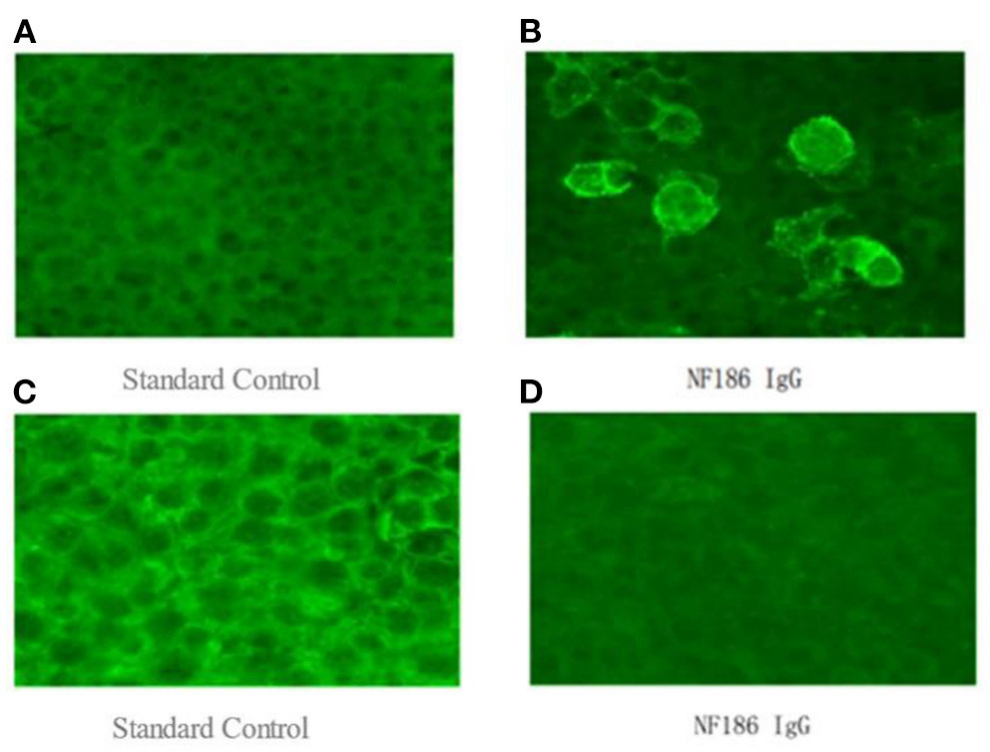
**(A,B)** a serum antibody test (Cell-based assay), which was examined on July 26, 2021, displayed anti-NF186 antibodies IgG 1:100. **(C,D)** the anti-NF186 antibodies IgG, which was examined on August 12, 2021, was negative after rituximab (RTX) treatment.

To further confirm the diagnosis, the B cell subset test *via* Flow cytometric immunofluorescence assay (FIFA) showed 7.83% CD20+ lymphocytes (see Supplementary Figure 1), and a serum antibody test (Cell-based assay, Figure 1) displayed anti-NF186 antibodies IgG 1:100. Then, we decided to let him stop taking oral prednisone and gave him rituximab with a total of 600 mg to suppress immunity along with other symptomatic and supportive treatments. The patient's condition was better than before. A week later, the B cell subsets examination showed 0.04% CD20+ lymphocytes (see Supplementary Figure 2) and the anti-NF186 antibodies IgG was negative (Figure 1).

### Outcome and Follow Up

**Figure 2:**
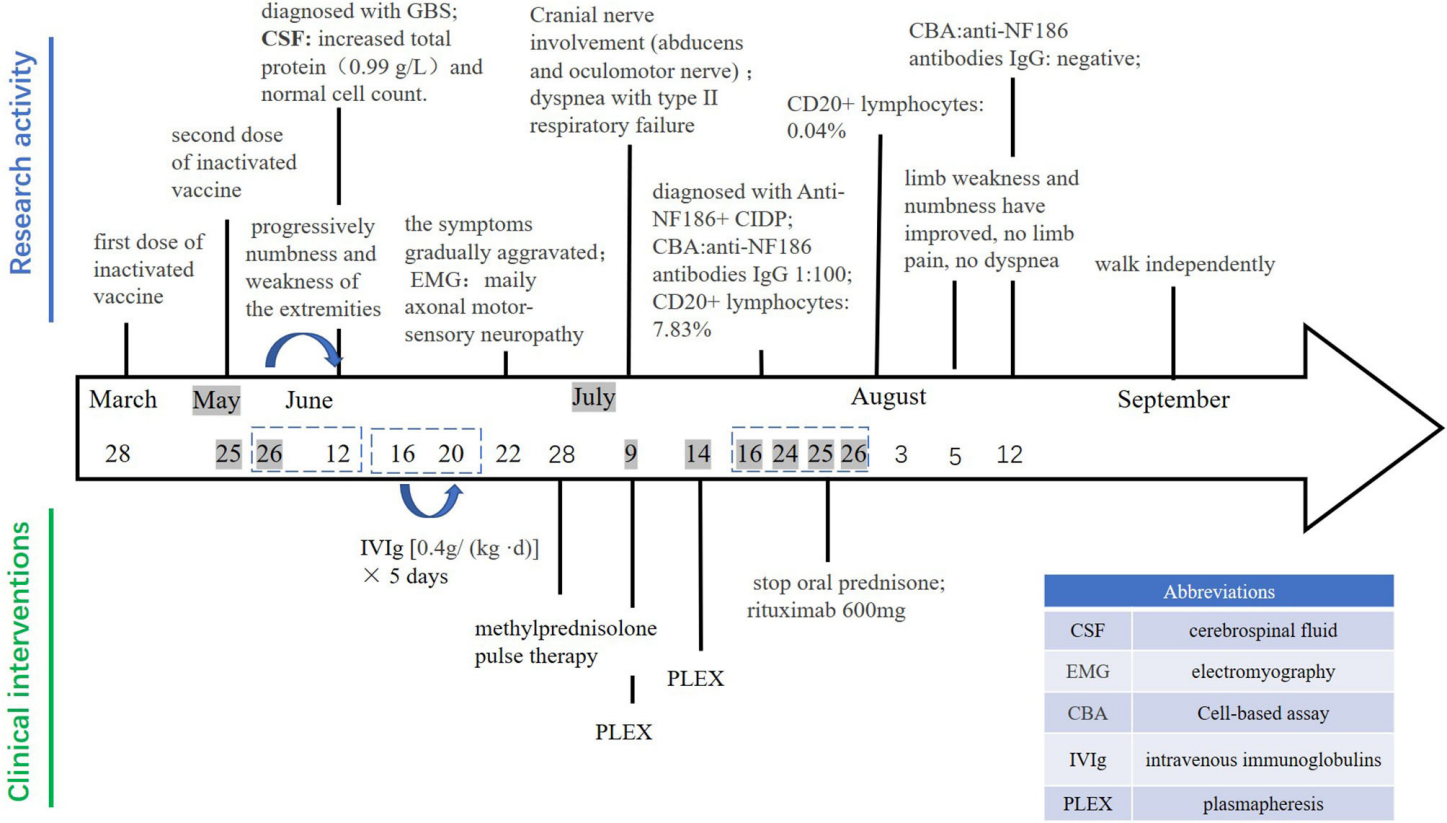
The timeline of clinical findings.

In August, the patient was diagnosed with immune-associated peripheral neuropathy, most likely to be anti-NF186+ CIDP. The patient has stopped using the ventilator, limb weakness and numbness have improved, no limb pain, and no dyspnea. A physical examination showed normal eye movement, symmetrical upper limb weakness (MRC grade 3/5 distally and 2/5 proximally), lower limb weakness (MRC grade 2/5 distally and 1/5 proximally), and areflexia. Muscle atrophy could be seen in the extremities, and the pathological signs were not elicited. In September, this patient was able to walk independently. Physical examination showed symmetrical upper and lower limb weakness (MRC grade 4/5 distally and 4/5 proximally, Figure 2).

## Discussion

The COVID-19 infection is a serious, complicated, and widespread disease; other than the respiratory symptoms, it is usually accompanied by a host of neurological complications. The wide spectrum of neurological complications includes cranial neuropathies with anosmia and dysgeusia, stroke, meningitis, and encephalitis. In addition, peripheral neuropathy, such as the GBS and CIDP, was reported in patients with COVID-19 infection (1–3). The GBS usually occurs following the infection, however, it has also been reported to occur after vaccination, surgery, or the administration of immune checkpoint inhibitors.

The safety profile of the inactivated vaccine for COVID-19 is good with commonly reported mild side effects such as pain at the injection site, allergic reactions on the skin, flu-like symptoms, headaches, fatigue, and so on (4). Post-vaccination neuropathies are rare events, and CIDP developing during the post-vaccination period is significantly unusual. To date, only two patients who developed CIDP after COVID-19 vaccination were reported, with a typical gradual onset of ascending lower limb weakness and sensory changes; one with facial involvement, another without (5). Therefore, our report extends the possible outcomes in patients who develop CIDP following COVID-19 vaccination and recommends that these patients need close monitoring after the acute phase to rule out the chronic evolution of the disease, which is critical for long-term treatment.

Our patient developed early-onset symptoms mimicking typical GBS with acute limb numbness and weakness after the inactivated COVID-19 vaccine. However, about 8 weeks later, his clinical symptoms continued to deteriorate, and cranial nerve involvement such as ophthalmoplegia appeared. Thus, after positive detection of NF-186 antibody, a diagnosis of NF186+ CIDP was confirmed. The CIDP is a common acquired immune-mediated peripheral neuropathy with strong clinical heterogeneity, including various clinical manifestations, and different responses to the same treatment. In recent years, early research found that autoantibodies against NF, contactin1 or contactin-associated protein 1 (Caspr) could be identified in ~10% of patients with CIDP. The pathology caused by these antibodies is called nodopathy-paranodopathy, unlike seronegative CIDP, which is no overt inflammation and demyelination and is characterized by dissection of myelin loops from axon at the paranode and subsequent axonal degeneration. In addition, patients with CIDP of this type typically respond poorly to IVIg but may benefit from plasmapheresis and rituximab (RTX) (6). These features are consistent with the clinical presentation of our patients. In our patient, electromyography displayed mainly axonal motor-sensory neuropathy and was accompanied by demyelinating damage. Moreover, he only responded to rituximab therapy.

In the blood test, our patient was found to have positive anti-nRNP/sm antibody, anti-nuclear antibody with 1:320 homogeneous + cytoplasmic granular type, and anti-double-stranded DNA. The complement C3 and C4 decreased. The urine routine showed a positive urine protein. Although the patient has no clinical manifestations, he met the diagnostic criteria for asymptomatic systemic lupus erythematosus (SLE). We speculate that the underlying disease SLE may induce the development of anti-NF186+ CIDP after vaccination.

We are admittedly aware of the lack of a biological marker to establish causality between anti-NF186+ CIDP and the vaccine. However, we cannot ignore the dramatic temporal association between receiving the vaccine and developing severe ophthalmoplegia, the prominent symptoms of dyspnea in a previously healthy male. Moreover, though the specific mechanism remains unknown, the hypothetical triggers for the pathogenesis of autoimmune disorders of the peripheral nervous system such as the Guillain–Barré syndrome (GBS) (1), acute inflammatory demyelinating polyneuropathy (AIDP), and CIDP (3, 7, 8) are viruses or viral vaccines. Some studies indicated it may be viewed as a result of the interaction between the susceptibility of the vaccinated subject and various vaccine components. Molecular mimicry is one of the implicated mechanisms, which refers to a significant similarity between certain pathogenic elements contained in the vaccine and specific human proteins (5, 9). Previous studies mentioned the potential for cross-reactivity between the coronavirus spike protein target produced by the messenger RNA (mRNA) vaccine and myelin basic protein (MBP) antigens. Moreover, adjuvants contained in vaccines (used mainly to increase the response to vaccination in the general population) may play a role in producing diverse autoimmune and inflammatory responses (10). The possible interactions between genetic predisposition, a history of other autoimmune conditions, the presence of adjuvants, and cross-reactivity between spike proteins and MBP antigens may all contribute to the genesis of peripheral neuropathy and require further investigation (9, 11).

## Conclusion

We report a rare case of anti-NF186+ CIDP after the second dose of inactivated COVID-19 vaccine. We attribute the occurrence of anti-NF186+ CIDP to the vaccine due to the temporal relationship and the lack of risk factors for CIDP in the patient. This report adds to the literature a possible rare side effect of a COVID-19 vaccine and contributes to the extremely limited literature on potential neurological side effects of inactivated vaccines. Healthcare providers should be aware of the possibility of post-vaccination CIDP. The patient had progressively aggravated limb weakness that was refractory to immunotherapy with pulse steroids and plasmapheresis, and with a dramatic response to RTX. This likely reflects an underlying autoimmune mechanism in the anti-NF186+ CIDP. Further research is needed to probe and study the exact mechanism at a more molecular level.

## Data Availability Statement

The original contributions presented in the study are included in the article/Supplementary Material, further inquiries can be directed to the corresponding author.

## Ethics Statement

Written informed consent was obtained from the individual(s) for the publication of any potentially identifiable images or data included in this article.

## Author Contributions

KH performed the systematic literature search and made figures. SW drafted the complete manuscript. HZ and PL collected the clinical data. LZ and LF revised the manuscript for important intellectual content. All authors gave the final approval of the final version to be published.

## Funding

This work was supported by the National Natural Science Foundation of China (Grant Nos. 81601139 and 81671299) and National Science Foundation of Hunan Province, China (Grant No. 2017JJ3500).

## Conflict of Interest

The authors declare that the research was conducted in the absence of any commercial or financial relationships that could be construed as a potential conflict of interest.

## Publisher's Note

All claims expressed in this article are solely those of the authors and do not necessarily represent those of their affiliated organizations, or those of the publisher, the editors and the reviewers. Any product that may be evaluated in this article, or claim that may be made by its manufacturer, is not guaranteed or endorsed by the publisher.
