# Supplementary material for: Case Report: Anti-NF186+ CIDP After Receiving the Inactivated Vaccine for Coronavirus Disease (COVID-19)
Source: Front Neurol. 2022 Mar 14;13:838222. doi: 10.3389/fneur.2022.838222 (PMC8964034; doi:10.3389/fneur.2022.838222)
Supplement: Supplementary file 1 [file Data_Sheet_1.docx]

10 Supplementary Material

10.1 Table 1: Auxiliary Examination

| Blood test | Result | Reference range |
| --- | --- | --- |
| Platelet count (109/L) | 113 | 120-350 |
| Percentage of neutrophils（%） | 78.8 | 40-75 |
| Albumin（g/L） | 30.3 | 40-55 |
| Globulin（g/L） | 40.6 | 20-40 |
| Erythrocyte sedimentation rate（mm/h） | 54.0 | 0-21 |
| Complement C3（mg/L） | 286.0 | 790.00-1520.00 |
| Complement C4（mg/L） | 35.8 | 100.00-400.00 |
| Immunoglobulin G（g/L） | 32.4 | 7.00-16.00 |
| Anti-nRNP/Sm | Weak positive | Negative（-） |
| Antinuclear antibody | 1:320 Homogeneous +cytoplasmic granular type | Negative（-） |
| Anti-double-stranded DNA | Positive（+） | Negative（-） |
| Anti-nucleolus | Positive（+） | Negative（-） |
| Antihistone | Weak positive | Negative（-） |
| Anti-Ro52 | Weak positive | Negative（-） |
| Urine test |  |  |
| Urine protein（g/L） | 1（2+） | Negative（-） |
| Occult blood（pcs/uL） | 200 (3+) | Negative（-） |
| Red blood cell count（pcs/HP） | 66.0 | 0-4 |
| White blood cell count（pcs/uL） | 10.56 | 0-5 |
| Normal morphology red blood cells（pcs/uL） | 50.16 | 0-6 |
| Abnormal red blood cells（pcs/uL） | 15.84 | 0-6 |

10.2 Figure 2


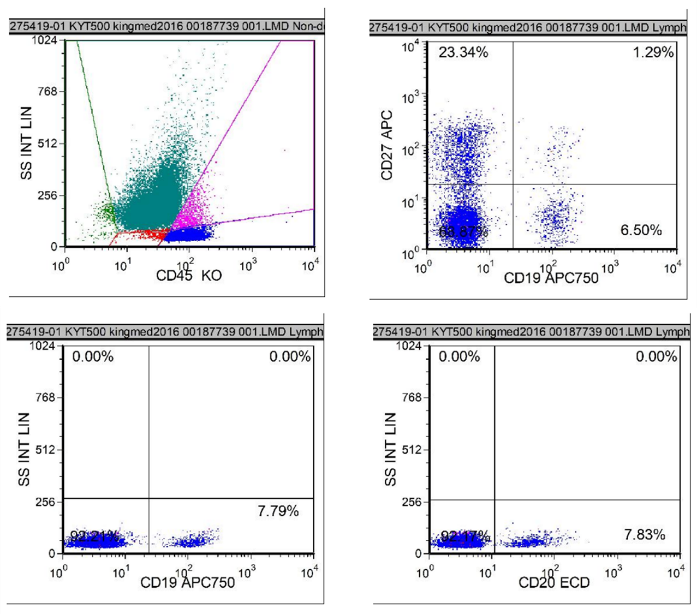


Figure 2. Before using rituximab, the B cell subsets examination was made on July 24, 2021，which showed CD20+Lymphocytes 7.83%.

10.3 Figure 3
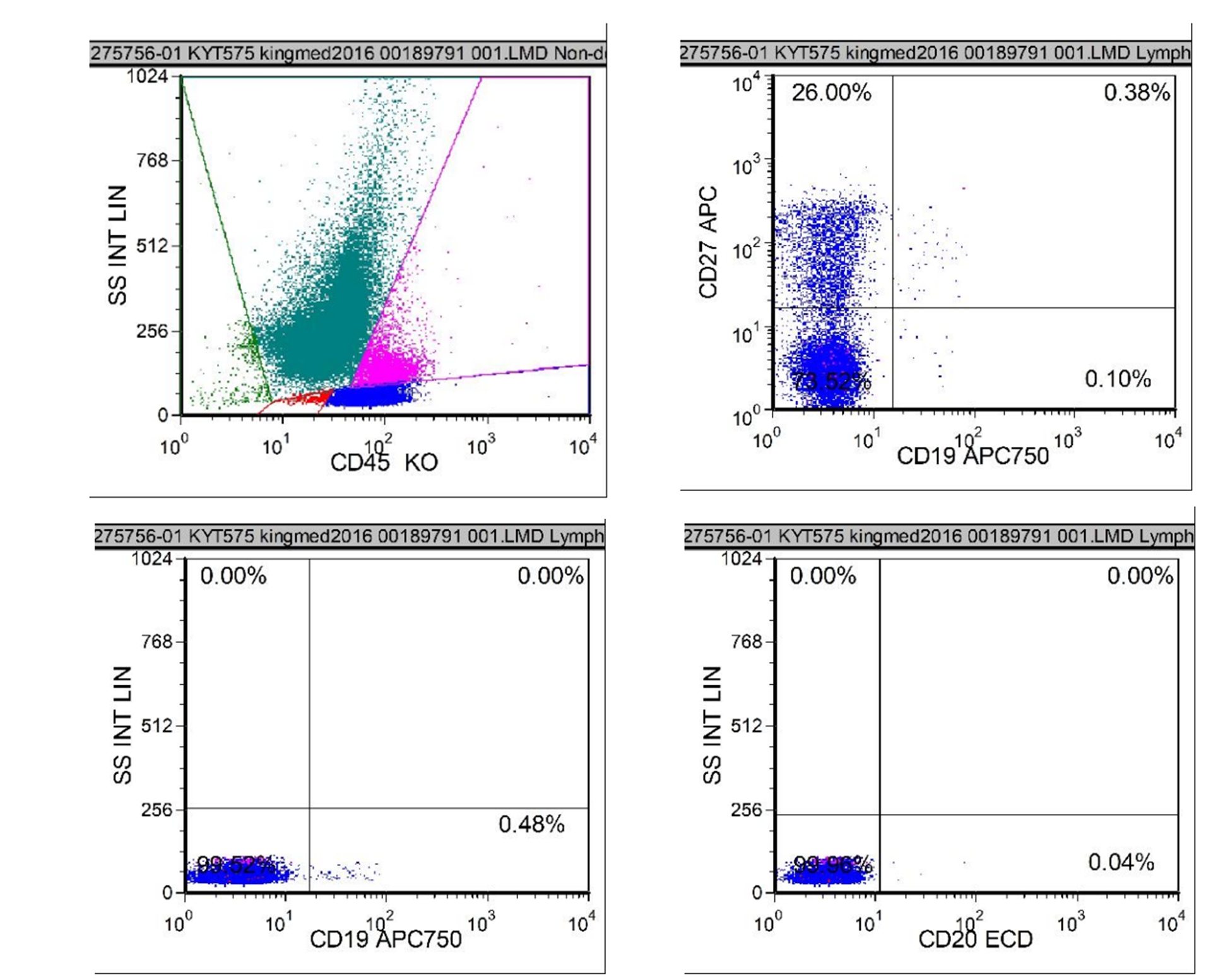


Figure 3. After using rituximab, the B cell subsets examination was made on August 3, 2021，which showed CD20+Lymphocytes 0.04%.
